# EHPnet: World Resources Institute: Climate, Energy, and Transport Program

**Published:** 2007-11

**Authors:** Erin E. Dooley

The World Resources Institute (WRI) works to advance scientifically based principles of sustainable and socially equitable development among international policy makers and institutions. One of its four central areas of work is climate protection as described at **http://www.wri.org/climate/**.

This gateway page provides recent highlights of activities including WRI analyses and statements to congressional and other federal bodies. Various sections detail international and U.S. action on climate protection, as well as WRI’s numerous programs and publications related to this topic. Other sections focus on the themes of technology options, green power and renewable energy use, and information and analysis tools.

The WRI has 17 climate protection programs. These range from the GHG [greenhouse gas] Protocol Initiative to the Vulnerability and Adaptation program, which seeks to reduce environmental and human impacts of climate change. Each program page describes the program’s aims and strategies, provides background and contact information, and links to publications and materials including press releases, reports, and workshop documents.

Two of the technology areas addressed by the WRI are carbon capture and sequestration (CCS) and sustainable transportation. The CCS program works to foster acceptance of these technologies among policy makers and the public, deploy a regulatory framework for CCS activities, and focus research on the most cost-effective methods. Currently 75 stakeholder organizations are involved in this initiative. The WRI Center for Sustainable Transport, known as EMBARQ, works mainly on urban issues. One of its successes has been the introduction of cleaner, more efficient buses in Mexico City, resulting in yearly carbon emission reductions there of almost 50,000 tons. On the basis of this outcome, a dozen more cities including Hanoi and Istanbul plan to duplicate this project.

